# Time Trends of Italian Former Smokers 1980–2009 and 2010–2030 Projections Using a Bayesian Age Period Cohort Model

**DOI:** 10.3390/ijerph110100001

**Published:** 2013-12-19

**Authors:** Giulia Carreras, Giuseppe Gorini

**Affiliations:** Cancer Prevention and Research Institute, via delle Oblate 2, Florence 50139, Italy; E-Mail: g.gorini@ispo.toscana.it

**Keywords:** smoking prevalence, former smokers, APC model, Bayesian analysis, Italy

## Abstract

This study aimed to describe past time trends of the prevalence of former smokers in Italy and to estimate prevalence projections using a Bayesian approach. An age-period-cohort (APC) analysis has been carried out in order to investigate the effect of the age, period and birth cohort on the prevalence of former smokers during 1980–2009. A Bayesian APC model with an autoregressive structure for the age, period and cohort parameters has been used to estimate future trends. Results showed that awareness of harm from smoking occurred at younger ages with each advancing cohort, and that women were more likely to attempt to stop smoking during pregnancies and breastfeeding, whereas men attempted to quit only when smoking-related diseases became evident. Projections of future trend recorded a further increase in the number of former smokers in future decades, showing an estimate of the “end of smoking” around years 2060 and 2055 in men and women, respectively. The application of the APC analysis to study the prevalence of former smokers turned out to be a useful method for the evaluation of past smoking trends, reflecting the effects of tobacco control policies on time and generations, and to make projections of future trend.

## 1. Introduction

Smoking is a major risk factor for many tumours and other chronic diseases, and reduces length and quality of life [1]. The implementation of tobacco control policies (TCP) recommended by the World Health Organization–Framework Convention on Tobacco Control (WHO-FCTC) is considered the leading primary prevention strategy to reduce smoking prevalence [2].

In Italy, several policies have been already implemented since the 1970s. A smoking ban in hospitals, schools, cinemas, and public transportations was introduced in 1975, followed by a smoking ban in front-offices of public administrations in 1995, and finally by a comprehensive smoking ban in all workplaces and in the hospitality sector in 2005 [3]. Tobacco advertising and promotion were almost totally banned in Italy since 1991, as in most European countries [4]. Moreover, the real price of cigarettes in 1990–2000 increased at an annual 3% rate [5]. In addition, several smoking cessation services were established since the end of the 1990s [3].

In response to all these efforts, smoking rates in Italy have progressively declined. Male smoking prevalence declined from 41.6% in 1986 to 29.5% in 2009, an average annual drop of 1.2%. Meanwhile, female smoking prevalence declined from 19.2% in 1986 to 17.0% in 1993, and stalled at that level [6]. At the same time, the prevalence of former smokers in men almost doubled from 15.2% in 1986 to 29.8% in 2009, whereas in women increased more than four times (from 3.4% to 15.8%). Prevalence of former smokers is a cumulated measure of smoking cessation, which accounts for those who stopped smoking in previous years. The proportion of annually quitters, however, was estimated to be very low in Italy: it was higher than 5% only in elderly persons and in women aged < 30 years, while in adults aged 30–49 and 50–59 cessations were about 2% and 3–5%, respectively. In addition, most of quit probabilities stalled from 1986 to 2009 [7].

The effect of smoking cessation policies may be highlighted by studying the prevalence of former smokers. Variations in prevalence of former smokers over time may be due to age, to a period effect, which is a reflection of changes in pro-tobacco efforts and smoking cessation policies in time, as well as to a cohort effect that may reflect different cumulative exposures to pro- and anti-tobacco efforts [8]. The age-period-cohort (APC) model is a descriptive tool for observations from a Lexis diagram that describes rates as a product of an age, a period, and a cohort effect in order to give an overview of the magnitude of the rates, the variation by age and time trends in the rates [9]. Such model is typically used in investigating vital rates with data from cancer registries or other disease registers. Two studies used the APC method to analyze smoking trends by applying the APC models to the prevalence of adolescent never smokers, and showing the effect of anti-tobacco programs to prevent adolescents from starting to smoke, primarily through a cohort effect [8,10].

This study investigated the effects of age, time period, and birth cohort on trends of Italian former smokers as a reflection of smoking cessation policies, by using the APC models on the trends of prevalence of former smokers. In addition, we predicted future trends of the prevalence of former smokers in Italy using a Bayesian APC approach, which incorporates prior information about smoothness on each time scale to reduce random variation and improve the precision of the projections [11]. 

The purpose is to use methods typically used in other contexts in order to advance our understanding of the trends of tobacco smoking and to provide data supporting evidence-based planning for more effective tobacco control. In addition, an estimate of the end of tobacco smoking may be given using this approach.

## 2. Methods

Data on smoking prevalence by gender and age were available from the Italian National Institute of Statistics (ISTAT) Multipurpose Surveys “Health conditions and access to health services” and “Aspects of daily living”. The two surveys are a surveillance system carried out almost every year between 1980 and 2009, and were based on similar scientific design providing comparable data on smoking habits over the years [12]. Sampling methods and data collection were already described elsewhere [6] and are briefly summarized herein. Surveys used a two-step sampling method: in the first step, about 900 municipalities were chosen, using different stratification criteria (demographic, geographic, and economic), and in the second step, a systematic sample of the families was selected within the municipality on the basis of demographic registries. Surveys were carried out on representative samples of the Italian population, and each survey was administered on average to 24,000 families and 54,000 persons distributed in about 850 Italian municipalities of different population sizes. Information was collected through questionnaires administered by professional interviewers. Smoking status was based on participant self-report as never, former, or current smoker [12]. Former smokers were those who reported smoking during their lifetimes but currently did not smoke. Prevalence of each smoking status in each survey was computed with sampling weight adjustment. Only former smokers over 25 years of age were considered, since the relative risks of death for smoking-related diseases are not discernable for those who quit smoking before that age [13].

Time trends of the prevalence of former smokers were evaluated through an APC analysis. Data were arranged in six 5-year periods (1980–1984, 1985–1989, 1990–1994, 1995–1999, 2000–2004, and 2005–2009) and twelve 5-year age groups (25–29 to 80–84 years). These age groups and calendar periods involved 17 overlapping 5-year cohorts identified by central year of birth, and defined using the relationship cohort = period − age [14,15] starting with 1900 and finishing with the cohort 1980. It should be noted that there are fewer observations for the earliest and most recent cohorts which may lead to less precision in the estimates of these cohorts.

We assessed the age, period, and cohort effects by means of generalized linear models assuming that the number of former smokers follows a Binomial distribution [14,15], since the more common Poisson distribution assumption of rare events is not appropriate for the prevalence of former smokers. Three models were used: the full APC model, age-period, and age-cohort (AC). In the APC analysis, there is the non-identifiable problem as age, period and cohort are linearly related [14,15]. In order to avoid the non-identifiability problem, we estimated the parameters of the full APC model using a two-step procedure [9]. In the first step, we fitted an AC model, using 1930 as the reference cohort in men and 1950 in women. The reference cohorts were chosen because they were the most exposed to cigarette smoking in Italy among men and women, respectively [12]. In the second step, we fitted a period effect to the residuals of the AC model by means of a Binomial model for the number of former smokers with the log of the fitted values from the AC model as offset. By this procedure, the standard deviations of the estimated values of the effects can be obtained assuming that the secular trend is related with the cohort effect [9]. Comparison between the nested models was evaluated by the changes in deviance (Δ-Dev) [9]. The Akaike Information Criterion (AIC) was also considered to identify the best-fitting model considering the best model the one with the lower AIC value [16]. Bayesian APC methods are often considered more appropriate for long-term projections due to the problem of random variation in classical parameter estimates of APC models [17] and consequently projections based on these estimates, particularly of the most recent cohort effects, are sensitive to this instability [11,18]. A Bayesian APC model smoothing the age, period and cohort effects and estimating the age-specific rates from their posterior distribution by Markov chain Monte Carlo (MCMC) techniques was used (see appendix) [11,18,19,20]. Constraints on first-order differences were set for the age, period and cohort effects (see appendix) [11,18,19,20]. For the analyses presented, posterior inferences were based on 1,000,000 iterations of the Gibbs sampler after a burn-in of 500,000 iterations was discarded. Convergence was assessed by using the Geweke statistic [21]. We carried out a sensitivity analysis on the prior distributions to evaluate the robustness of the results (appendix). In order to evaluate the goodness of fit the observed prevalence of former smokers cumulated by age was plotted together to the fitted one. The statistical software used for the analyses was R [22] and JAGS [23].

## 3. Results and Discussion

**Figure 1:**
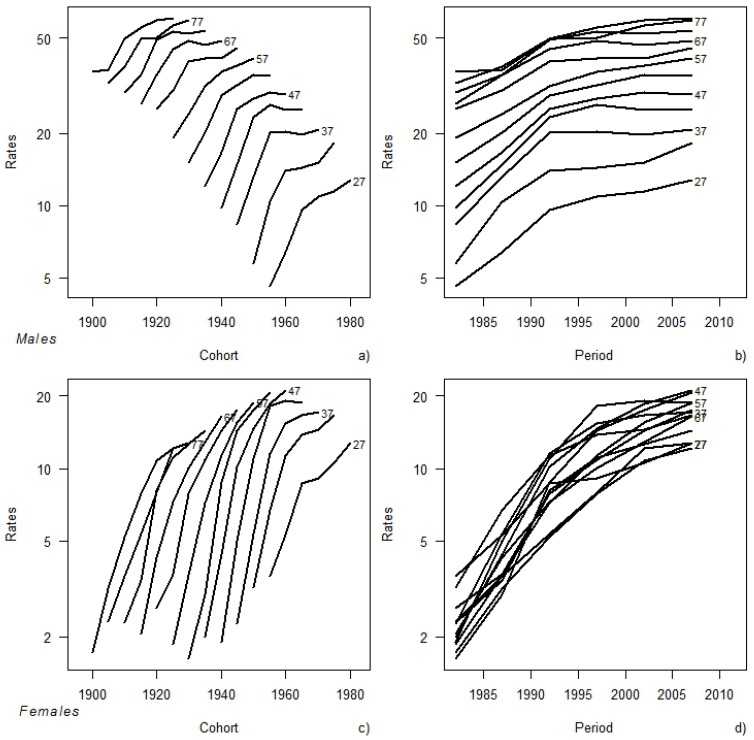
Age-specific prevalence of former smokers (**a**) by birth cohort and (**b**) by time period in males. Age-specific prevalence of former smokers (**c**) by birth cohort and (**d**) by time period in females.

Figure 1 shows the patterns of age-specific rates by birth cohort and time period for both genders. Younger cohorts have higher prevalence rates for all ages both in males and in females (Figure 1a,c). In fact, as an example, men former smokers aged 47 years are 12.0% and 29.2% in the 1982 and 2007 birth cohorts, respectively. The rates appear to increase steadily with age with no evidence of downturn in males (Figure 1a,b). This was not true for females among whom the proportion of former smokers is similar for all age groups, with prevalence values smaller than that recorded in males (Figure 1c,d). An increasing trend for all age groups was observed mainly up to 1992 in males, whereas in females the rates showed an increased trend in the whole time period.

In the APC analysis we obtained similar results for both genders (Table 1). The APC model showed the lowest AIC value, thus both cohort and period effects should be taken into account. The Δ-Dev was higher for the AC model than for the AP one. Thus, the period effect was more important than the cohort effect in describing prevalence of former smokers. An APC analysis restricted to the period 1995–2009 showed however for both genders an AIC value for the AC model comparable to the APC one, revealing that cohort effect was the most important factor to explain the variability in the prevalence of former smokers during that period.

**Figure 2:**
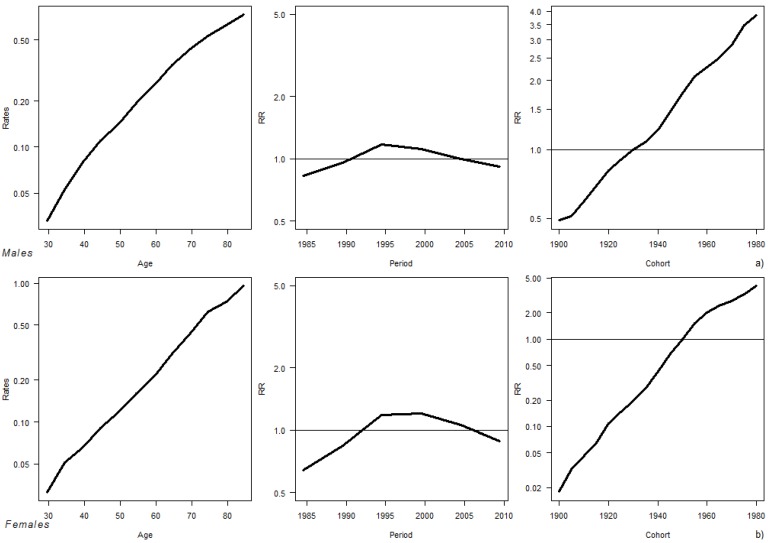
Age, period, and cohort effects from the APC model over the period 1980–2009 for (**a**) males and (**b**) females.

In Figure 2 the age, period, and cohort effects from the APC model over the period 1980–2009 for males and females are reported. For both genders the age effect grew with the increasing of age, the period effect was almost negligible reaching a peak in 1992–2002, and the cohort effect showed an increasing trend in the whole period. For both males and females confidence intervals of the estimated effects resulted very narrow.

**Table 1:** APC model assessment for prevalence of former smokers in Italy in periods 1980–2009, and 1995–2009 for males and females.

	1980–2009			1995–2009		
Males	AIC	DEV	Δ-Dev	AIC	DEV	Δ-Dev
APC	627	43,391	-	168	5276	-
AP	1642	115,482	72,091	687	23,494	49,635
AC	5,689	402,846	359,455	181	5,734	392
Females						
APC	559	38,613	-	196	6,293	-
AP	2,829	199,872	161,259	1338	46,301	40,007
AC	5,398	382,230	343,618	239	7,791	1,497

Bayesian projections showed a global convergence with the Geweke statistic over 1.96 for 3 and 8 parameters out of 132 for males and females, respectively. Sensitivity analyses confirmed the stability of posterior prevalence values over different values of the prior hyper parameters, producing posterior prevalence values that fell inside the credible interval. In particular, the maximum difference among posterior projections was of 0.44 and 2.32 points percentage in males and in females respectively, in both cases observed for the last year of projections. 

**Figure 3:**
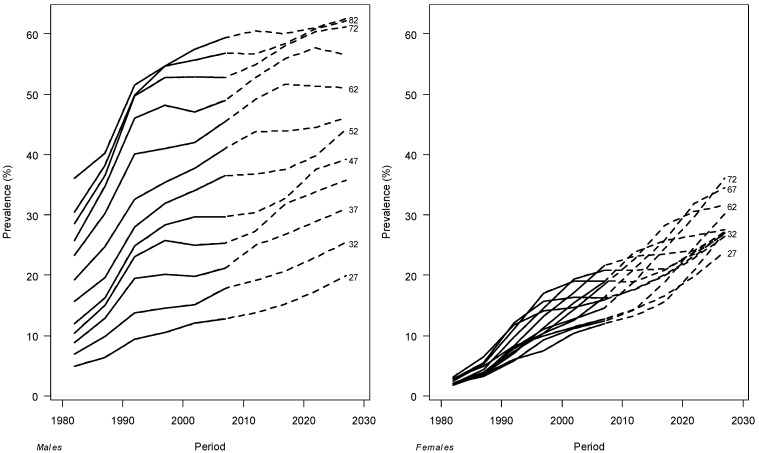
Age-specific prevalence of former smokers by period, fitted in 1980–2009 (solid line) and projected for 2010–2030 (dotted line) for males and females.

Age-specific former-smoker prevalence fitted and projected by cohort using the APC Bayesian model with the 1980–2009 period as basis for projections are reported in Figure 3. The former smokers in men will continue to show an age-gradient being higher for the older age groups and lower for the younger. The prevalence of former smokers for older men will reach 62.5% of the population in 2030. The prevalence for younger men will reach 19.9%. The prevalence of former smokers in females showed similar values and future trend for all age-classes reaching on average 36.1% of the population in 2030. In females projections showed very fast rates of increase. However, estimates for the last period were highly uncertain. In fact, while the width of the 95% credible intervals for the fitted rates indicated precise estimates, on the other hand projections showed a progressive widening of the credible intervals (data not shown). 

**Figure 4:**
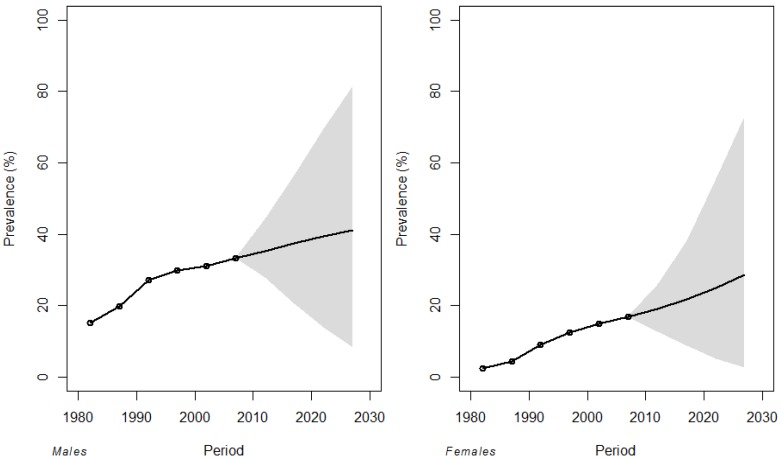
Fitted and projected prevalence of former smokers cumulated by age (25–85 years) with 95% credible intervals by period for males and females. Dots are the observed cumulated prevalence.

In Figure 4 the fitted and projected prevalence curves cumulated by age (25–85 years) with the 95% credible intervals are reported. Also the observed prevalence of former smokers is reported in Figure 4, indicating a good model fit. Former smokers in 2030 will be around 42.7% of men and 32.1% of women. As in the age-specific estimates, also in this case projections for the last period were highly uncertain with credible intervals rapidly widening (Figure 4).

We described time trends of the prevalence of former smokers and projected trends for the next decades in Italy. In 1980–2009 younger cohorts showed higher prevalence values for all ages in both genders. Rise in prevalence occurred earlier in life, as for more recent cohorts. This is likely to reflect awareness of harm from smoking that occurred at younger ages in each advancing cohort [24]. The prevalence increased with age in males, and was similar for all age-groups in females. This is probably due to the fact that women were more likely to attempt to stop smoking at younger age during pregnancies and breastfeeding. On the contrary, men attempted to quit only when smoking-related diseases became evident [7]. 

The APC analyses showed that the period effect reached its maximum in 1992–2002, as an effect of the laws implemented in that period, such as smoking ban on TV advertisement (1991), smoking ban on public administrations (1995), and rise in cigarettes taxes (from 1990). At the same time, the cohort effect showed a progressive continuous increase since the first generation, suggesting that awareness of harm from smoking progressively became stronger. The analysis restricted to the time period 1995–2010 revealed an increasing importance of the cohort.

Projections of the cumulated prevalence showed that former smokers will reach 42.7% of men and 32.1% of women in 2030 (Figure 4). These estimates should be considered together with the gender specific prevalence of non smokers and smokers, being the population mutually composed by smokers, non smokers, and former smokers. The prevalence of non smokers in males showed an increasing trend in the last decades reaching the value of 41.2% in 2012. In females, even if the prevalence is still higher than the males one (66.3% in 2012), the opposite trend was observed [12]. By linearly projecting gender specific non smokers prevalence, it is possible to roughly estimate the “end of smoking” in Italy. Under these assumptions the “end of smoking” will be around 2060 in men and 2055 in women (Table 2), a more optimistic estimate respect to a recent English research by Citigroup that estimated the Italian “end of smoking” in 2091 [25]. These estimates are however based on a linear regression from UK smokers prevalence data. By making the same computations using Italian specific data on the last decade, the “end of smoking” will occur in 2046.

**Table 2:** Projections on the smokers prevalence (%) for males and females.

Year	Prevalence (%)	
	Males	Females
2012	23.44	14.55
2017	22.10	14.24
2022	19.37	12.06
2027	16.96	9.58
2032	14.72	7.05
2037	11.73	6.37
2042	9.09	4.58
2047	6.45	2.80
2052	3.80	1.01
2057	1.16	0.00
2062	0.00	0.00

The novelty of this work is the use of APC methods, which are usually used for the study of trends in deaths rates, to study trends in the prevalence of former smoker, and to make projections in a Bayesian perspective.

This study has some limitations. First, prevalence projections resulted highly uncertain. The credible intervals encompass both uncertainty associated with the choice of the model and uncertainty associated with projecting beyond the range of the data. This is necessarily reflected by the rapidly increasing width of the intervals as the length of the projection increases [18]. Second, the use of MCMC algorithms in the field of latent Gaussian models, as regression models for longitudinal data, has been criticized in terms of both computational time and mixing due to strong dependencies of parameters in the posterior distribution, and of weak identifiability. Several alternatives were proposed to overcome these problems, as integrated nested Laplace approximations [26]. However our application did not exhibit convergence problems with the use of MCMC, even if computational times were rather long. A third limitation is that further measures to improve cessation rates, such as, a further increase in cigarette taxes and promoting smoking cessation strategies (reimbursement of cessation treatments, setting up an active quit line for smokers, and promoting widespread use of cessation counselling among health professionals) could be implemented in the period for which we made projections [27].

## 4. Conclusions

In conclusion, this study showed a constant increase in prevalence of former smokers in Italy since 1980s, and regarding all generations and age-groups. Projections of future trend recorded a further increase in the number of former smokers in future decades, showing an estimate of the “end of smoking” around years 2060 and 2055 in men and women, respectively.

## Appendix—Autoregressive APC Model for Projections of the Prevalence of Former Smokers

Following Breslow and Clayton [19], Berzuini and Clayton [20] and Bray [11,18] we specify a Gaussian autoregressive prior model in the forward direction to smooth effects on each timescale and to extrapolate period and cohort effects.

According to this model each point (except the first two on each scale) is predicted by linear extrapolation from its two immediate predecessors, plus a random error from a normal distribution with mean 0. The first two parameters for age, period and cohort effects are given non-informative priors. The distributions for the α_1_,…, α_A_ age, β_1_,…, β_P_ period, and γ_1_,…, γ_C_ cohort effects, can be defined as follows:

The precision of the normal distributions can be represented as a hyperparameter for which the prior distribution reflects prior belief concerning the smoothness of the parameters. In our analyses we use non-informative hyperprior distributions so that the hyperparameters are estimated solely from the data:

The count of former smokers *d_ap_* in each age group *a* and period *p* follow a binomial distribution with parameters *p_ap_* and *n_ap_*, where *n_ap_* is the known population size of age group *a* and period *p*, and *p_ap_* is the unknown prevalence. Fitted and projected prevalence of former smokers are then obtained through a recombination of the smoothed age, period and cohort effects according to:

We carried out a sensitivity analysis on the prior distributions, to evaluate the robustness of the results. In particular, we changed the mean of the normal distributions for the first two parameters for age, period, and cohort from 0 to 0.01. Then we changed the shape and inverse scale parameters of the Gamma distributions for the age, period and cohort one at time from 0.001 to 0.005.
